# Redesigning library orientation for first-year medical students during the pandemic

**DOI:** 10.5195/jmla.2021.1190

**Published:** 2021-07-01

**Authors:** Andy Hickner, Drew Wright, Loretta Merlo, Janna S. Gordon-Elliott, Diana Delgado

**Affiliations:** 1 alh4014@med.cornell.edu, Weill Cornell Medicine, New York, NY; 2 drw2004@med.cornell.edu, Weill Cornell Medicine, New York, NY; 3 lamerlo@med.cornell.edu, Weill Cornell Medicine, New York, NY; 4 jsg2005@med.cornell.edu, Weill Cornell Medicine, New York, NY; 5 did2005@med.cornell.edu, Weill Cornell Medicine, New York, NY

**Keywords:** undergraduate medical education, library orientation, remote learning

## Abstract

**Background:**

Prior to 2020, library orientation for first-year medical students at Weill Cornell Medicine took the form of an on-site treasure hunt competition. Due to the COVID-19 pandemic, the orientation for the MD class of 2024 was shifted to an all-virtual format. This shift mandated a full redesign of the library orientation.

**Case Presentation:**

The Samuel J. Wood Library sought to preserve the excitement and fun of the treasure hunt in the new virtual format. The competition was redesigned as a Zoom meeting using breakout rooms, with library faculty and staff serving as team facilitators. Tasks were rewritten, shifting the focus from the library's physical spaces to its virtual services and online resources. The redesigned orientation was evaluated using two data sources: a postsession survey of student participants and a debriefing of the library employees who participated. Student evaluations were positive, while the faculty and staff provided numerous suggestions for improving future virtual orientations.

**Conclusions:**

A successful virtual library orientation requires careful preparation, including testing the competition tasks, full rehearsal with library facilitators, and a thoughtful approach to technology and logistics. We have chosen to share the materials we developed for other academic health sciences libraries that may wish to take a similar approach to their own virtual orientations.

## BACKGROUND

The Samuel J. Wood Library & C.V. Starr Biomedical Information Center (hereafter referred to as “library”) serves Weill Cornell Medicine (WCM), including Weill Cornell Medical College, Cornell University's medical school. Currently, WCM provides training and education for 406 medical students (including sixty-one MD/PhD students). From 2006 to 2019, the library conducted first-year medical student (M1) orientations as a “treasure hunt” [1, 2]. Students were divided into teams and raced around the library from station to station. Each station was staffed by two library employees, who provided clues necessary to complete the treasure hunt. The goal of the activity was to introduce students to library services, policies, space, and employees in a way that was fun and informative. At the end of the treasure hunt, each team completed a brief online quiz. First, second, and third place prizes were awarded to the first three teams to complete the treasure hunt and submit the quiz with a perfect score.

Orientation provides an opportune time to introduce key policies. On the other hand, orientation week is a less-than-ideal time for students to learn more complex information and research skills, since they are absorbing large amounts of information for a week or more [3]. Consequently, the learning goals of the treasure hunt were secondary to the goals of providing a welcoming experience for students and beginning to establish relationships between students and library employees. The treasure hunt is students' first encounter with the library, where student-library relationships typically begin; according to one of the authors who has worked at the library for nearly four decades, some of these connections have extended beyond their four years in the program. Its design utilized hands-on and team learning, taking advantage of and enhancing students' receptiveness to getting to know one another and their new learning environment.

During the COVID-19 pandemic, due to campus and public health mandates, the orientation for the MD class of 2024 was shifted to an all-virtual format. Moreover, due to the pandemic, only a small portion of the class was living nearby and expected to be utilizing the physical library for at least the first semester. The shift to an entirely virtual setting required a full redesign of the library orientation. However, the year of 2020 was not the first time the library had updated the M1 orientation to incorporate new technologies; for example, beginning in 2016, the library successfully introduced the use of iPads and QR codes into the treasure hunt [2, 4].

## CASE PRESENTATION

The goal of the virtual redesign was to preserve the excitement and fun of the treasure hunt, while introducing students to the library in a way that would be relevant to them regardless of whether they would be physically accessing the library within the first semester. Considering the virtual format and the students' short-term expected mode of utilizing library services, this 2020 update required extensive revision of both the learning outcomes and the structure of the activity.

To deemphasize in-person engagement with the library, the learning outcomes related to the physical space were either removed or shifted to the library director's presentation beforehand. The presentation covered information such as identification badge requirements to enter the library, how to access the campus wireless network, computers, printing, scanning, and how to schedule an appointment at the Library and Information Technology Services front desk. In contrast to the treasure hunt, the virtual activity started with a clinical vignette to ground it in a real-life context. Set in the pediatric clerkship, the vignette involved a fictional consultation with a young child and a mother who expressed hesitancy about scheduled immunizations. The activity was created as a Qualtrics quiz, which can be viewed at https://library.weill.cornell.edu/sites/default/files/vaccination_hesitation.pdf. The final version of the activity materials is available on eCommons, the Cornell University institutional repository, at https://hdl.handle.net/1813/76942 [5].

Library employees participated in brainstorming sessions during the activity redesign, identifying relevant tools and tasks that would provide a comprehensive introduction to resources and services offered by the library, the Medical Center Archives, and the Myra Mahon Patient Resource Center. Some of the newly developed tasks tested students' retention of essential information introduced in the library director's presentation, while others provided hands-on practice using the library's electronic resources, services, and/or website. Table 1 lists the tasks and their associated learning objectives.

**Table 1:** Learning objectives and tasks

Learning objective	Task
Locate and use the library's collections and electronic resources	Using one of the library's licensed online textbook collections to answer a background question
	Obtaining the full text of an article using PubMed and the library's link resolver
	Using the library's subject guides
	Using the Medical Center Archives
Locate patient education materials	Using the Myra Mahon Patient Resource Center website and Medline Plus to locate patient education materials
Use research and scholarly communications tools	Identifying potential predatory journals
	Using WCM's research-focused discovery tool VIVO (http://vivo.med.cornell.edu)
Use library services	Testing students' awareness of interlibrary loan services
	Using the library's chat service

According to cognitive load theory, learners can only actively process a limited amount of new information at any time [6]. As such, the quiz was carefully written in order to minimize unnecessary cognitive load, with each task on a separate page. Appropriate scaffolding information was included in the text of each task to focus students' attention on the tasks themselves (e.g., definitions of concepts such as predatory journals and background questions). Instructions for students were incorporated into the quiz. To minimize unnecessary confusion, we sought to make both the instructions and the correct answers as clear and specific as possible. The full activity was first piloted with several library employees and revised based on their feedback.

The orientation took place entirely within a single Zoom meeting set up by the activity leader, in this case, the education and outreach librarian. Ten teams were created for the 106 students, with each team consisting of ten to eleven students and a library employee who served as the team's facilitator. A total of fourteen library employees were involved in the orientation, including the eleven facilitators (one team had two facilitators), the activity leader, a response grader, and the library director. Each team was preassigned to its own breakout room. Careful configuration of the Zoom meeting was crucial. For example, participants were required to authenticate using the college's single sign-on in order to ensure they could be automatically transferred to their assigned breakout rooms at the appropriate time. Each team was asked to designate a team captain who was responsible for screen-sharing, completing the Qualtrics activity for the team, and submitting the completed activity. Where possible, automatic scoring of correct answers was configured in order to minimize the amount of manual scoring required.

In addition to the Qualtrics quiz, extensive effort was invested in preparing the facilitators and developing step-by-step instructions for them. Microsoft Teams was used as a “backchannel” to allow the library facilitators to communicate during the activity.

After the library director's presentation, the activity leader introduced the activity and provided detailed instructions. The activity leader turned on breakout rooms, and the teams were automatically sent to their assigned rooms. In breakout rooms, everyone briefly introduced themselves, and the teams selected their captains. After all teams completed introductions and selected captains (approximately five minutes), the activity leader sent the Qualtrics link to all rooms, and the teams began the activity. As soon as a team finished the activity, the facilitator messaged the Microsoft Teams backchannel. The librarian assigned to scoring reviewed the hand-scored items for each submission. The teams that finished early were asked to complete the postorientation survey. Immediately after verifying the first- and second-place teams, the grader announced the winners in the backchannel. The activity leader then closed the breakout rooms, and all teams were returned to the main room, where the activity leader announced the winning teams and made concluding remarks.

One additional change to the activity was that only two teams were awarded prizes, as opposed to three team winners in the past. This was due to austerity measures affecting the hospital and medical college caused by the pandemic.

### Student assessment

The virtual activity took significantly less time than the face-to-face treasure hunt. From the beginning of the introductory remarks by the activity leader to the announcement of the winning teams took approximately thirty-five minutes. Teams completed the activity in an average of just under nine minutes, compared to twenty to thirty minutes for the treasure hunt. All groups successfully completed all tasks except for one group, which provided an incorrect answer for the task involving patient education materials.

As in previous years, a postsession survey [7] was administered to students, including two questions about students' experience with the orientation. Although larger proportions of students reported that the orientation was fun/very fun or mostly/very informative in 2020 compared with 2019 (Table 2), these differences were not statistically significant (Fisher's exact test [8], *p*>0.05). Other questions included items regarding student interest in using various library resources and services, the frequency with which they used the Internet for biomedical research, and their level of experience using various databases. The survey instrument is available in the Supplemental File.

**Table 2:** Student assessment data

Overall, how fun was the orientation?	Percent of students in 2019 (n=85)	Percent of students in 2020 (n=88)
Not fun	2.4	0
Somewhat fun	10.6	5.6
Mostly fun	9.4	6.7
Fun	24.7	31.5
Very fun	52.9	56.2

Overall, how informative was the orientation?	Percent of students in 2019	Percent of students in 2020
Not informative	0	0
Somewhat informative	3.5	1.1
Neutral	2.4	1.1
Mostly informative	32.9	35.2
Very informative	61.2	62.5

At the end of the survey, students were invited to provide free text feedback. Ten students provided feedback, all of which was positive. Students described the session as “engaging,” “fun,” and “one of the best of the week.”

### Facilitator assessment

The small-group facilitators participated in a debriefing two days after the orientation in order to discuss what worked well and to identify improvements for future orientations. Facilitators provided extensive and detailed feedback and identified several problems. There was confusion in some groups over the official start time for the competition. The volume of Teams messages made it easy for facilitators to miss important messages and questions from the activity leader. Facilitators' comfort with the Zoom interface varied, with at least one facilitator requiring assistance with basic tasks during the event. This demonstrates the importance of conducting a full technical rehearsal in breakout rooms ahead of time. Finally, while the competition aspect increased student engagement, there were trade-offs. As one facilitator noted, “I don't think they read the story past the main page. Instead, they just read the action item in bold. Providing a copy of the activity by email afterward might help the students who don't read as quickly to understand what was going on, and what services are available. Some quieter students may have gotten left in the virtual dust!”

Facilitators reported finding the facilitator guide helpful. They noted that it was helpful to have a back-up facilitator and that two facilitators per team would be ideal.

## DISCUSSION

Our virtual adaptation of the medical student “treasure hunt” orientation to the library and library services was found to be feasible and acceptable by student-participants and staff.

Below, we share recommendations based on our experience. In general, prepare for the unexpected. For example, hours before the orientation it was discovered that there was an unplanned outage of the textbook platform used in the first task; that task was consequently deleted at the last minute.

### Technological support

The facilitators' comfort level and experience using Zoom ranged widely. We recommend scheduling a full technical rehearsal well in advance of the activity so that facilitators can practice each step. If possible, the institution's information technology staff should be on hand to assist facilitators when they need help in their breakout rooms or to troubleshoot other complications during the session.

### Dissemination of information

We suggest providing all instructions in the large-group introduction, including slides to visually reinforce instructions. Questions about the process should also be addressed in the large group introduction. This ensures every group gets the same information in a clear, unrushed way and reduces the burden on small group facilitators. Once teams are sent to breakout rooms, they should be allotted a specific amount of time for brief introductions and selection of the team captain.

It may be helpful to send the content of the activity to all students after the session is over to ensure that they can integrate what they learned regardless of their speed of information processing (the activity may go quickly, with the pace determined by those participants who process this kind of information rapidly). Reintroduction to what was learned during subsequent points of their training may also be useful, as repetition and exposure in a variety of contexts may help reinforce the information. As an example, we are currently developing a series of educational modules in the college's learning management system. We plan to reuse the instructional content that we developed for the virtual orientation in these modules, including learning outcomes and practice exercises.

Our report adds to existing literature on the use of games in academic library orientations [9] and its utility in engaging students by leveraging their sense of competition [10]. Our student feedback data is consistent with that reported by other academic libraries that have implemented virtual orientations: that it students found them effective and helpful [11–13] and in some cases even preferred them over in-person orientation [14, 15]. Because we were able to compare student data from both in-person and online cohorts using the same survey instrument, our report helps fill the evidence gap reported by Dana Ingalls in her 2015 review of literature on virtual library orientations, who called for more quantitative studies on their efficacy [16].

Previous published reports describing library orientations for first-year medical students reflect the pre-pandemic status quo of in-person orientations [7, 17–19]. Ours is one of a handful of recent case reports of the experiences of academic health sciences libraries adapting instruction to the virtual format to the COVID-19 pandemic [20, 21].

Any virtual library orientation will need to reflect the specific programs and setting in which it is developed. A total of fourteen library employees were involved in our orientation. We felt that the smaller student-to-employee ratio was beneficial in terms of establishing a sense of personal connection with students. There is evidence to correlate this hypothesis elsewhere in the literature. For example, Gall (2014) found that students who participated in a synchronous, in-person library orientation were more likely to contact the library for assistance than those assigned to an asynchronous online orientation [22]. Likewise, Gotschall et al. (2021) reported that according to student evaluations of another virtual orientation during the pandemic, “many students would have preferred a one-on-one interaction with their librarian via Zoom's breakout rooms” [20]. For libraries with a smaller number of employees, less labor-intensive alternatives have been reported, including a virtual scavenger hunt using a mobile application [23], augmented reality [24], or asynchronous video [25]. While our format may not be feasible for all institutions due to differences in resources and settings, we believe that sharing our format, materials, and lessons learned will assist other libraries seeking ways to maintain a high-quality orientation experience for students in the post-COVID-19 world. This report and others to come may lay the groundwork for a more rigorous investigation into best practices for virtual library orientations in medical school settings.

## Data Availability

Data associated with this article are available in eCommons, Cornell University's institutional repository, at https://hdl.handle.net/1813/102703.

## SUPPLEMENTAL FILES

**Library Assessment Survey**
