# Supplementary material for: Redesigning library orientation for first-year medical students during the pandemic
Source: J Med Libr Assoc. 2021 Jul 1;109(3):497–502. doi: 10.5195/jmla.2021.1190 (PMC8485957; doi:10.5195/jmla.2021.1190)
Supplement: Supplementary file 1 — Library Assessment Survey [file jmla-109-3-497-s01.docx]

Library Assessment Survey

Start of Block: Welcome

Q1 Thank you for participating in the Samuel J. Wood Library’s orientation that introduced you to the many resources and services that are available to you. These resources and services will be available to you throughout your educational experience at Weill Cornell Medicine.
Please take this brief survey to help us understand if you found the orientation valuable. The Library would also like to use this opportunity to learn a little bit more about you to help us meet your information needs in the coming years. The average survey completion time is less than 10 minutes. All responses are optional.
This study has been approved by the Weill Cornell Institutional Review Board (1401014726)

End of Block: Welcome

Start of Block: Library Assessment

Q35 Library Assessment

Q2 Now that you have been introduced to the Samuel J. Wood Library, what are you most interested in using for the coming year (choose 3)?

- Online journals (1)
- Online books (2)
- Physical materials (3)
- SMARTDesk (4)
- Research reference (5)
- Special topics consultations (6)
- 24/7 Study Room (afterhours) (7)
- Historical photographs and documents from the Medical Center Archives (10)
- Other: (9) ________________________________________________

Q3 Overall, how informative was the orientation?

- Not Informative (1)
- Somewhat Informative (2)
- Neutral (3)
- Mostly Informative (4)
- Very Informative (5)

Q4 Overall, how fun was the orientation?

- Not Fun (1)
- Somewhat Fun (2)
- Mostly Fun (3)
- Fun (4)
- Very Fun (5)

End of Block: Library Assessment

Start of Block: Information Resources

Q36 Information Resources

Q6 On average, how often do you use the Internet for biomedical research?

- Once a week (1)
- Multiple times a week (2)
- Once a day (3)
- Multiple times a day (4)

Q7 Which of these biomedical-related databases have you used? Please select any that apply.

- BIOSIS (1)
- Ovid Medline (2)
- PubMed (3)
- Scopus (4)
- Web of Science (5)
- None of the above (6)
- Other (7) ________________________________________________

Q8 Do you use more than trial and error to find information on the Internet?

- Yes (1)
- No (2)

Q9 What criterion or criteria is best for evaluating Internet-based biomedical information? Please select any that apply.

- Accuracy (1)
- Authority (2)
- Availability (3)
- Findability (4)
- Legibility (5)
- Objectivity (6)
- Relevancy (7)
- Timeliness (8)
- Usability (9)

Q10 In general, which learning format do you prefer?

- Self-paced online (1)
- Real-time web-based seminar (2)
- One-on-one in-person instruction (3)
- Small in-person group instruction (eight or less) (4)
- Large in-person group instruction (nine or more) (5)

Q11 Please rank your preferred technologies for accessing online biomedical information:

|  | Least Preferred (1) | Less Preferred (2) | More Preferred (3) | Most Preferred (4) |
| --- | --- | --- | --- | --- |
| Smartphones (1) |  |  |  |  |
| Tablets (iPad, Surface, etc) (2) |  |  |  |  |
| Laptops (3) |  |  |  |  |
| Desktops (4) |  |  |  |  |

Q12 What would be your preferred way of seeking assistance with finding research-related information?

- In-person visit to the library (1)
- Virtual session via Zoom (2)
- Not interested in either (3)

Q13 A “personal librarian” service would link each WCMC student with one librarian who would guide information seeking and generally help with research. How likely is it that you would enroll in this service if it were available through the WCMC Library?

- Unlikely (1)
- Somewhat Unlikely (2)
- Neutral (3)
- Somewhat Likely (4)
- Very Likely (5)

End of Block: Information Resources

Start of Block: Health Informatics

Q37 Health Informatics

Q14 Have you ever seen an electronic health record (EHR)?

- Yes (1)
- No (2)

Q15 If so, which EHR?

- Epic (1)
- eClinicalWorks (2)
- NextGen (3)
- Allscripts/Eclipsys (4)
- Do not know (5)
- Other (6) ________________________________________________

Q16 Have you ever seen a personal health record (PHR)?

- Yes (1)
- No (2)

Q17 If so, which PHR?

- EPIC Connect (1)
- HealthVault (2)
- Sunrise Patient Portal (3)
- Do not know (5)
- Other: (4) ________________________________________________

Q18 What is your level of interest for learning more about EHRs and PHRs?

- Not at all interested (1)
- Slightly interested (2)
- Moderately interested (3)
- Very interested (4)
- Extremely interested (5)

Q19 What level of expertise do you have with each of the following types of software:

|  | Don't know what that is (1) | Novice (2) | Proficient (3) | Expert (4) | Not sure (5) |
| --- | --- | --- | --- | --- | --- |
| Spreadsheets (i.e. Microsoft Excel) (1) |  |  |  |  |  |
| Databases (i.e. Microsoft Access, MySQL) (2) |  |  |  |  |  |
| Computer programming or scripting (i.e. Python, JAVA, PHP,R) (3) |  |  |  |  |  |
| Statistical software packages (i.e. R, SPSS, STATA, SAS) (4) |  |  |  |  |  |

Q20 What is your level of interest for learning more about the above types of software?

|  | Not at all interested (1) | Slightly interested (2) | Moderately interested (3) | Very interested (4) | Extremely interested (5) |
| --- | --- | --- | --- | --- | --- |
| Spreadsheets (i.e. Microsoft Excel) (1) |  |  |  |  |  |
| Databases (i.e. Microsoft Access, MySQL) (2) |  |  |  |  |  |
| Computer programming or scripting (i.e. Python, JAVA, PHP,R) (3) |  |  |  |  |  |
| Statistical software packages (i.e. R, SPSS, STATA, SAS) (4) |  |  |  |  |  |

End of Block: Health Informatics

Start of Block: Bioinformatics

Q38 Bioinformatics

Q23 Please indicate your level of expertise with any of the following bioinformatics research tools

|  | Don't know what that is (1) | Novice (2) | Proficient (3) | Expert (4) | Not sure (5) |
| --- | --- | --- | --- | --- | --- |
| Specialized bioinformatics databases (e.g. NCBI, KEGG, UniProt) (4) |  |  |  |  |  |
| Open source analysis libraries (e.g. bioconductor) (1) |  |  |  |  |  |
| Proprietary bioinformatics software (e.g. Ingenuity Pathway Analysis, Lasergene) (11) |  |  |  |  |  |

Q24 What is your level of interest for learning more about these research tools?

|  | Not at all interested (1) | Slightly interested (2) | Moderately interested (3) | Very interested (4) | Extremely interested (5) |
| --- | --- | --- | --- | --- | --- |
| Specialized bioinformatics databases (e.g. NCBI, KEGG, UniProt) (4) |  |  |  |  |  |
| Open source analysis libraries (e.g. bioconductor) (11) |  |  |  |  |  |
| Proprietary bioinformatics software (e.g. Ingenuity Pathway Analysis, Lasergene) (12) |  |  |  |  |  |

Q40 What kind of electronic lab notebooks have you used (select all that apply)?

- LabArchives (1)
- SciNote (2)
- Jupyter notebooks (3)
- Other (4) ________________________________________________
- I have not used an electronic lab notebook (5)

Q25 What is your level of interest for learning more about electronic laboratory management tools such as Lab Archives?

- Not at all interested (1)
- Slightly interested (2)
- Moderately interested (3)
- Very interested (4)
- Extremely interested (5)

End of Block: Bioinformatics

Start of Block: Demographics

Q39 Demographics

Q26 Weill Cornell Educational Track

- Medical Student (1)
- Graduate Student (2)
- MD/PhD Student (3)

Q27 Age

- 20 or younger (1)
- 21-25 (2)
- 26-30 (3)
- 30-34 (4)
- 35 or older (5)
- Decline to answer (6)

Q31 Highest Degree Achieved

- BS (1)
- BA (2)
- Master's (3)
- MD (4)
- PhD (5)

Q33 May we contact you for any future library-related research efforts?

- Yes (1)
- No (2)

Display This Question:

If May we contact you for any future library-related research efforts? = Yes

Q32 Weill Cornell E-mail (i.e. cwid2013@med.cornell.edu)

________________________________________________________________

Q34 Please share any other comments you may have:

________________________________________________________________

________________________________________________________________

________________________________________________________________

________________________________________________________________

________________________________________________________________

End of Block: Demographics
